# Molecular Characterization of *FT* and *FD* Homologs from *Eriobotrya deflexa* Nakai forma *koshunensis*

**DOI:** 10.3389/fpls.2016.00008

**Published:** 2016-01-22

**Authors:** Ling Zhang, Hao Yu, Shunquan Lin, Yongshun Gao

**Affiliations:** ^1^State Key Laboratory for Conservation and Utilization of Subtropical Agro-bioresources, College of Horticulture, South China Agricultural UniversityGuangzhou, China; ^2^Department of Biological Sciences and Temasek Life Sciences Laboratory, National University of SingaporeSingapore, Singapore

**Keywords:** loquat, flowering, floral transition, *FT*, *FD*

## Abstract

In angiosperms, regulation of flowering is a vital process for successful reproduction. To date, the molecular mechanism of flowering is well-studied in the model plant, *Arabidopsis*, in which key genes such as *FLOWERING LOCUST* (*FT*) or *FD* have been identified to regulate flowering. However, the flowering mechanisms are still largely unknown in fruit trees like loquat. To this end, we first cloned one *FT-* and two *FD-*like genes from the loquat (*Eriobotrya deflexa* Nakai f. *koshunensis*) and referred to as *EdFT*, *EdFD1*, and *EdFD2*, respectively. Phylogenetic analysis has shown that EdFT, EdFD1, and EdFD2 are conserved during the evolution process. *EdFT* is mainly expressed in reproductive tissues (e.g., flower buds, flowers, and fruits), while *EdFD1* and *EdFD2* are mainly expressed in apical buds including leaf buds and flower buds. EdFT is localized in the whole cell, while EdFD1 or EdFD2 is localized in the nucleus. Ectopic expression of *EdFT*, *EdFD1*, and *EdFD2* in *Arabidopsis* results in early flowering. In addition, we have also revealed that the EdFT interacts with both EdFD1 and EdFD2. Overall, these data suggest that the EdFT, EdFD1, and EdFD2 are the functional homologs of FT and FD, respectively, which might act together to regulate loquat flowering through a similar mechanism found in *Arabidopsis*.

## Introduction

In angiosperms, flowering, known as a key process of the transition from vegetative to reproductive growth, is crucial for plant reproductive success. The initiation of flowering is coordinately regulated via the integration of external and internal signals, including photoperiod, temperature, plant age, and gibberellic acid (Wellmer and Riechmann, 2010; Song et al., 2013). During the past few decades, extensive studies in the model plant *Arabidopsis* have identified different floral signaling pathways, where different types of flowering time genes act in response to various factors (Amasino, 2010; Wellmer and Riechmann, 2010; Andres and Coupland, 2012). Notably, these signaling pathways finally converged on a floral pathway integrator, FLOWERING LOCUS T (FT), which serves as the component of the long-sought florigen (Pennisi, 2007; Turck et al., 2008; Song et al., 2013; Blumel et al., 2015).

*FT* mRNA is expressed in leaves and its protein moves to the shoot apex through the phloem to promote flowering (Corbesier et al., 2007; Liu et al., 2013a). FT encodes a phosphatidylethanolamine-binding protein (PEBP), whose family consists of three phylogenetically distinct groups: FT-like proteins, TERMINAL FLOWER1-like (TFL1) proteins and MOTHER OF FT AND TFL1-like (MFT) proteins (Pin and Nilsson, 2012; Wickland and Hanzawa, 2015). *MFT*-like genes are in the basal clade and are considered as the ancestors of *FT* and *TFL1*-like genes. In *Arabidopsis*, the PEPB family comprises six genes including *FT*, *TFL1*, *MFT*, *TWIN SISTER OF FT* (*TSF*), *BROTHER OF FT* (*BFT*), and *ARABIDOPSIS THALIANA RELATIVE OF CENTRORADIALIS* (*ATC*; Matsoukas et al., 2012; Kim et al., 2013). TSF, a member of the FT-like clade, is also produced in the phloem companion cells, and then transported to the apex to trigger flowering (Jang et al., 2009). In contrast to *FT*-like genes, *TFL1*, *BFT*, and *ATC* have antagonistic function in flowering and are classified into the *TFL1*-like clade (Yoo et al., 2010; Huang et al., 2012).

FT is transported to the shoot apex, where it interacts with FD to induce flowering in *Arabidopsis* and *Oryza sativa* (Hayama et al., 2003; Tsuji et al., 2013). *FD*, encoding a bZIP transcription factor, is mainly expressed in the shoot apex during floral induction (Wigge et al., 2005). In *Arabidopsis*, FT and FD are found to interact with each other both *in vivo* and *in vitro*, and the FT–FD complex directly activates the floral meristem identity genes like *APETALA1* (*AP1*) through binding to its promoter (Abe et al., 2005; Wigge et al., 2005). The mutation in *FD* results in late flowering and suppresses the early flowering phenotype of *FT* overexpression lines, suggesting that FD mediates the function of FT in regulating flowering (Wigge et al., 2005). In addition to the extensive studies in *Arabidopsis*, FT and FD have also been identified in other plants such as tomato (Pnueli et al., 2001; Lifschitz and Eshed, 2006), pea (Sussmilch et al., 2015), kiwifruit (Varkonyi-Gasic et al., 2013), rose (Randoux et al., 2014), strawberry (Mouhu et al., 2009), poplar (Bohlenius et al., 2006; Hsu et al., 2006; Tylewicz et al., 2015), Satsuma mandarin (Endo et al., 2005; Nishikawa et al., 2007), and apple (Kotoda et al., 2010; Guitton et al., 2012). Functional characterization of these genes has revealed that they have a conserved role in regulating flowering.

Loquat (*Eriobotrya* Lindl.) is a subtropical evergreen fruit tree in the apple subfamily (maloideae; rosaceae). Unlike other rosaceous fruits, such as apple, strawberry and peach, that are widely cultivated in the world, loquat is mainly distributed throughout Southeast Asia and Mediterranean countries and regions. It is famous as a tasty fruit that has rich nutrition and ripens at off seasons, and also as a medicinal plant that contains many pharmaceutical compounds (Hong et al., 2008). The cultivated loquat (*Eriobotrya japonica* Lindl.), which is the only edible specie of genus *Eriobotrya*, blooms in fall or early winter (Lin et al., 1999). Besides the cultivated loquat, there are a lot of wild species in *Eriobotrya* (Lin, 2007) that demonstrate different flowering time. For example, *E. deflexa* Nakai flowers in spring (Wu and Peter, 2003), which is similar to the other Rosaceae plants. In China, we have observed that the same loquat variety grown in different places may blossom and yield fruits in different seasons. The similar phenomenon is also observable in other fruit trees. Thus, we aim to study the flowering mechanisms of loquat to understand the molecular basis of various flowering patterns. This will help us to determine how to optimize loquat yield in different climatic areas. So far molecular studies on loquat flowering are still limited, and have mostly focused on the cultivated loquat. Two *LEAFY* (*LFY*) and two *TFL1* orthologous genes have been cloned from the cultivated loquat (Esumi et al., 2005). In addition, one *EjAP1* has been isolated from ‘Zaozhong No. 6.’ Ectopic expression of this gene in the *Arabidopsis ap1-1* mutant rescues sepal and petal development (Liu et al., 2013b), indicating the functional convervation of floral meristem identity genes between loquat and *Arabidopsis*.

In this study, we cloned one *FT-* and two *FD*-like genes from *E. deflexa* Nakai f. *koshunensis*, and named them *EdFT*, *EdFD1*, and *EdFD2*, respectively. Sequence analyses, expression studies, and functional characterization of these loquat genes in *Arabidopsis* suggest that these three genes may evolve to have some unique functions in loquat development in addition to similar roles in regulating flowering time to their *Arabidopsis* orthologs.

## Materials and Methods

### Plant Materials and Growth Conditions

Wild loquat (*E. deflexa* Nakai f. *koshunensis*) was selected for this research. Loquat trees were grown under natural conditions in the loquat germplasm resource preservation garden, South China Agricultural University, Guangzhou, China. The wild type *Arabidopsis thaliana* ecotype Col was used for gene transformation. *Nicotiana benthamiana* was grown for transient expression. *Arabidopsis* and tabacco were grown under long-day conditions (16 h light/8 h dark) at 22°C.

### Gene Isolation and Sequence Analysis

The full-length coding sequences of *EdFT*, *EdFD1*, and *EdFD2* were amplified from the cDNA prepared from loquat leaves using Phusion DNA Polymerase (Thermo, USA). Subsequently, the PCR products were cloned into pGEM-T easy vector (Promega, USA). The primers used for gene cloning were listed in **Supplementary Table S1**. Amino acid sequences were aligned using ClustalX and BioEdit. Phylogenetic analyses were performed using MEGA by the Neighbor-Joining (N-J) method with 1000 bootstrap replications. The identity of the nucleotide and amino acid between *EdFD1* and *EdFD2* was analyzed using DNAMAN V6.0. The sequences of the *EdFT* and two *EdFD* genes was deposited in GenBank, the accession numbers are KU319433 (EdFT), KU319434 (EdFD1), and KU319435 (EdFD2).

### Vector Construction

To construct *35S:EdFD1/2-6HA*, the *EdFD1/2* coding sequences were amplified and introduced into pGreen-35S-6HA vector (Hou et al., 2014). To produce *35S:EdFT/EdFD1/EdFD2-GFP*, *EdFT/EdFD1/EdFD2* coding sequences were cloned into pGreen-35S-GFP (Lee et al., 2012). For the bimolecular fluorescence complementation (BiFC) assay, *35S:EdFT-cYFP* and 35S:*nYFP-EdFD1/2* were constructed by cloning *EdFT* and *EdFD1/2* into pGreen-35S-cYFP/nYFP (Hou et al., 2014). All constructed vectors were confirmed by sequencing. All of the primers used for vector construction were listed in **Supplementary Table S2**.

### *Arabidopsis* Transformation

The constructed vectors were introduced into *Agrobacterium tumefaciens GV3101::psoup* and then transformed into *Arabidopsis* Col using the floral dip method (Zhang et al., 2006). Transgenic lines were screened on soil by Basta.

### Transient Expression in *Nicotiana benthamiana*

For observing the subcellular localization of EdFT, EdFD1 and EdFD2, and the BiFC analysis, *Agrobacterium*-mediated transient transformation in *N. benthamiana* leaves was performed as previously described (Sparkes et al., 2006). After transformation, fluorescent signals were observed under the fluorescence microscope (Observer.D1, Zeiss, Germany) or the confocal microscope (510 Meta, Zeiss, Germany).

### Gene Expression Analysis

The total RNA was isolated by EASYspin Plus plant RNA extraction kit (aidlab, China), and the cDNAs were synthesized using PrimeScript^TM^ RT reagent Kit with gDNA Eraser (TaKaRa, Japan). Quantitative real-time PCR (qPCR) was performed using iTaq^TM^ universal SYBR Green Supermix (Bio-Rad, USA) in the LightCycler 480 (Roche). The specificity of primers was confirmed by melting curve analysis and sequencing. The PCR efficiency was measured by standard curve in the LightCycler^®^ 480 SW 1.5 software. And primers with specific amplification and efficiency about 2.0 were applied to test primer efficiency. Gene expression levels were normalized against loquat *ACT4* (JX089589). Semi-quantitative reverse transcription PCR (RT-PCR) was used for detecting exogenous gene expression in *Arabidopsis* overexpression lines. *TUB2* (AT5G62690) was amplified as an internal control. The primers used were shown in **Supplementary Table S3**. All the expression analysis were applied with three technical replicates and two biological replicates.

### Data Analysis

The significance of the differences between data was evaluated with the Student *t*-test. Calculations were carried out using Microsoft Excel software.

## Results

### Identification of *FT* and *FD* Orthologs from *E. deflexa* Nakai f. *koshunensis*

**FIGURE 1:**
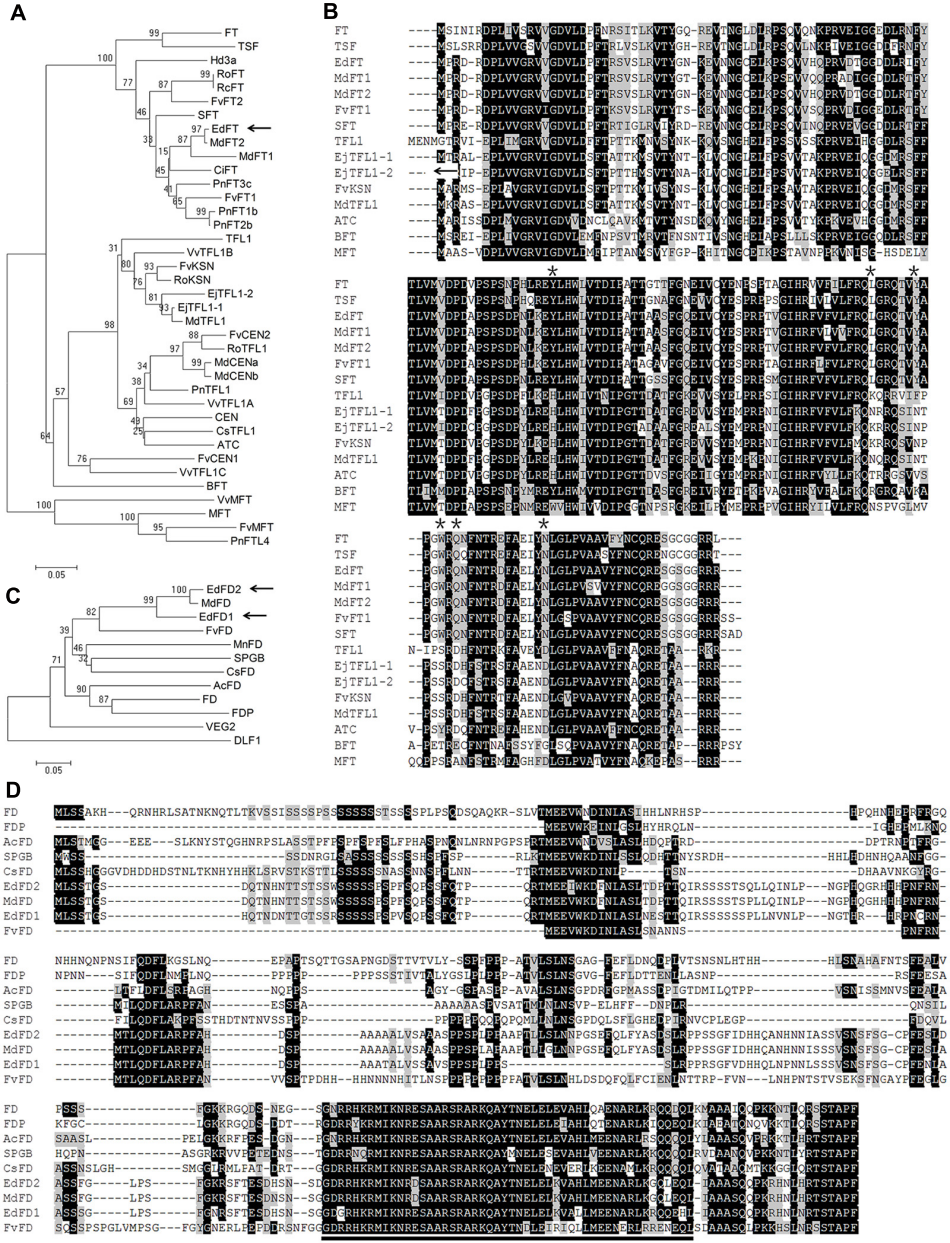
**Sequence analysis of EdFT and EdFDs.** **(A)** Phylogenetic analysis of plant PEBP family proteins. The accession number of each gene is as follows: *FT* (*Arabidopsis thaliana*, AT1G65480), *TFL1* (AT5G03840), *TSF* (AT4G20370), *MFT* (AT1G18100), *BFT* (AT5G62040), *ATC* (AT2G27550), *SFT* (*Solanum lycopersicum*, AY186735), *EjTFL1-1* (*Eriobotrya japonica*, AB162045), *EjTFL1-2* (AB162051), *MdFT1* (*Malus*× *domestica*, AB161112), *MdFT2* (AB458504), *MdTFL1* (AB052994), *MdCENa* (AB366641), *MdCENb* (AB366642), *FvFT1* (*Fragariavesca*, JN172098), *FvFT2* (XM_004297225), *FvCEN1* (XM_004308059), *FvCEN2* (XM_004291562), *FvKSN* (HQ378595), *FvMFT* (XM_004299493), *RoFT* (*Rosa luciae*, FM999826), *RoTFL1* (FM999796), *RoKSN* (HQ174211), *RcFT* (*Rosa chinensis*, FR729041), *CiFT* (*Citrus unshiu*, AB027456), *PnFT1b* (*Populus nigra*, AB161109), *PnFT2b* (AB161108), *PnFT3c* (AB161107), *PnTFL1* (AB369067), *PnFTL4* (AB181241), *Hd3a* (*Oryza sativa*, AB052941), *CEN* (*Antirrhinum*, S81193), *CsTFL1* (*Chrysanthemum seticuspe*, AB839767), *VvTFL1A* (*Vitis vinifera*, DQ871591), *VvTFL1B* (DQ871592), *VvTFL1C* (DQ871593), *VvMFT* (DQ871594). Arrows indicate the *EdFT* gene. **(B)** Amino acid sequence alignment of several plant PEBP family proteins showed in **(A)**. Amino acid residues in black and gray represent 100 and 50% similarity respectively. The asterisks indicate the critical amino acid positions. **(C)** Phylogenetic analysis of plant FD proteins. The accession number of each gene is as follows: *FD* (AT4G35900), *FDP* (AT2G17770), *AcFD* (*Actinidia chinensis*, JX417425), *SPGB* (*Solanum lycopersicum*, EF136919), *MnFD* (*Morus notabilis*, XM_010115002), *VEG2* (*Pisum sativum*, KP739949), *CsFD* (*Chrysanthemum seticuspe*, AB839769), *DLF1* (*Zea mays*, NM_001112492), *MdFD* (*Malus* × *domestica*, XM_008339760), *FvFD* (*Fragaria vesca*, XM_004289026). Arrows indicate the *EdFD1/2* genes. **(D)** Amino acid sequence alignment of several plant FD proteins showed in **(C)**. Amino acid residues in black and gray represent 100 and 50% similarity, respectively. The bZIP domain is underlined.

Since *FT* and *FD* in *Arabidopsis* and their orthologs in other plant species have shown important roles in regulating flowering time, we first isolated one *FT-* and two *FD*-like genes from *E. deflexa* Nakai f. *koshunensis*, and named them *EdFT*, *EdFD1*, and *EdFD2*, respectively (**Supplementary Figure S1**). *FT* encodes a protein of PEBP family. Phylogenetic analysis of EdFT and six *Arabidopsis* PEBP family proteins showed that EdFT was classified into the FT clade (**Figure 1A**). Although the amino acid sequence of FT shares high similarity to that of TFL1, they play antagonistic roles in regulating flowering time in *Arabidopsis*. Substitution of single critical amino acid residue leads to the function conversion between FT and TFL1, and *vice versa* (Hanzawa et al., 2005; Ho and Weigel, 2014). Sequence alignment revealed that the critical amino acid residues in EdFT were identical to those in FT rather than those in TFL1 (**Figure 1B**; asterisks indicate the critical sites). In addition, EdFT showed the highest sequence similarity to MdFT homologs (99% identity to MdFT2, and 94% to MdFT1), which were isolated from *Malus × domestica* in the same subfamily of Maloideae as loquat.

FD is a bZIP protein and interacts with FT to activate the floral meristem identity genes in *Arabidopsis* (Abe et al., 2005; Wigge et al., 2005). In loquat, we also isolated two *FD*-like genes, *EdFD1* and *EdFD2*. They shared high sequence similarity to each other, with 83.93% identity at the nucleotide level and 84.31% identity at the amino acid level. Phylogenetic analysis showed that EdFD1 and EdFD2 were similar to the reported FD proteins, especially those from Rosaceae (**Figure 1C**). The amino acid sequence alignment also showed that EdFD1 and EdFD2 contained a bZIP domain, which is conserved in other FD proteins (**Figure 1D**).

### Tissue-Specific Expression Patterns of *EdFT* and *EdFDs* in Loquat

**FIGURE 2:**
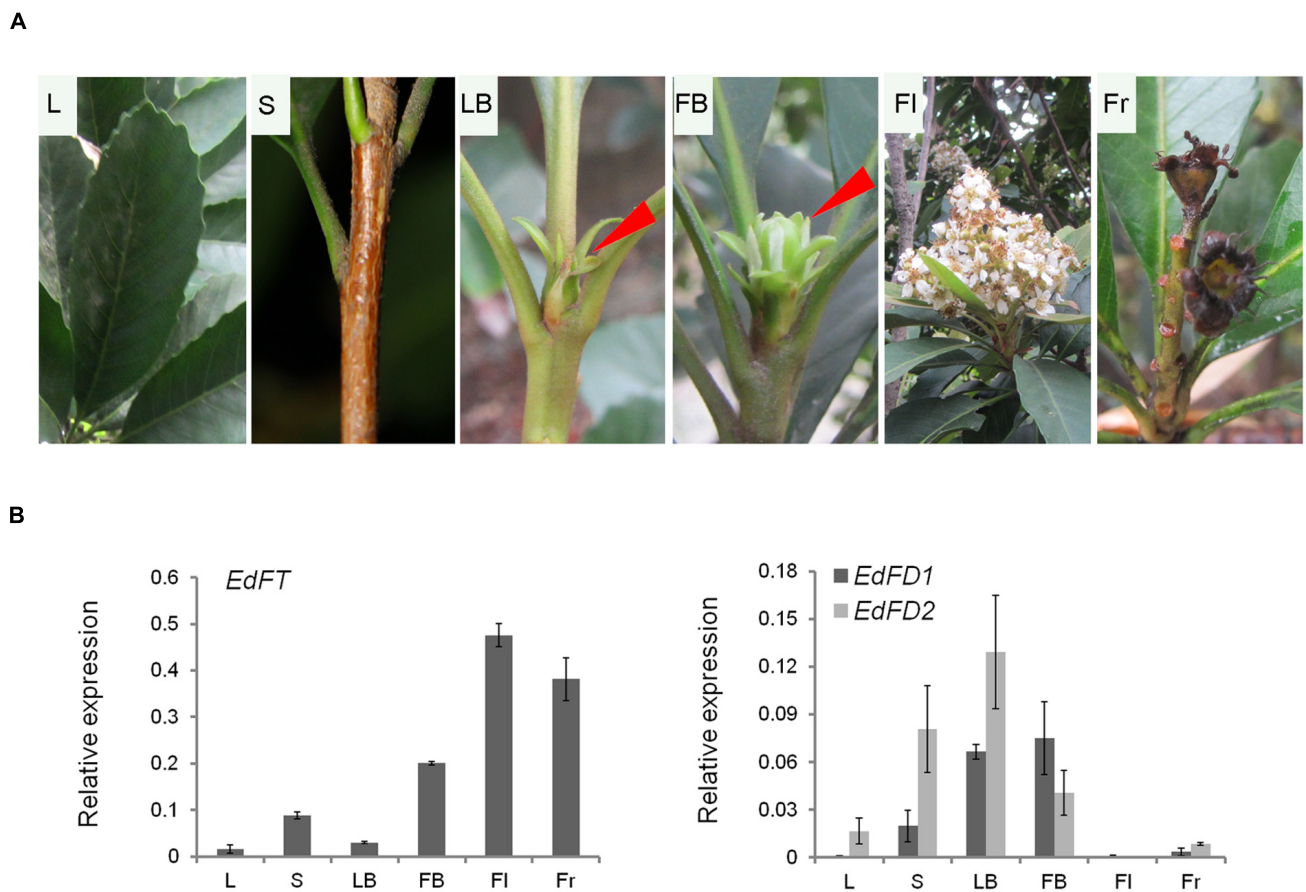
**Tissue-specific expression of *EdFT*, *EdFD1*, and *EdFD2* in loquat.** **(A)** Different tissue phenotype from the loquat *Eriobotrya deflexa* Nakai f. *koshunensis.* **(B)** Relative expression of *EdFT*, *EdFD1*, and *EdFD2* in different tissue showed in **(A)**. Loquat *ACT4* gene serves as an internal control. L, leaf; S, shoot; LB, leaf buds; FB, flower buds; Fl, flower; Fr, fruit.

To understand the potential function of *EdFT* and *EdFDs* in loquat, we examined the expression pattern of *EdFT* and *EdFDs* using qPCR in various tissues of *E. deflexa* Nakai f. *koshunensis*, including leaves, shoots, leaf buds, flower buds, flowers, and fruits (**Figure 2A**).

*EdFT* was highly expressed in flower buds, flowers and fruits, which was similar to the expression patterns of its orthologs in apple and kiwifruit (Kotoda et al., 2010; Varkonyi-Gasic et al., 2013). Unlike *FT* in *Arabidopsis*, which is highly expressed in leaves (Corbesier et al., 2007), *EdFT* expression levels were low in leaves (**Figure 2B**). This suggests that *EdFT* might also participate in the development of flowers and fruits in addition to its expected function in regulate flowering.

*EdFD1* and *EdFD2* were expressed with a comparable pattern. Their expression levels were high in leaf buds and flower buds, but low in leaves, flowers, and fruits (**Figure 2B**). In *Arabidopsis*, *FD* is mainly expressed in the shoot apex before the floral transition, while during the floral transition, FD interacts with the uploaded FT protein in the shoot apex to co-regulate the subsequent floral meristem development (Wigge et al., 2005). Interestingly, we found that *EdFT*, *EdFD1*, and *EdFD2* were all expressed at relatively high levels in flower buds (**Figure 2B**), implying that EdFD1/EdFD2 might interact with EdFT in flower buds to co-regulate flower development in loquat.

### Subcellular Localization of EdFT and EdFDs

**FIGURE 3:**
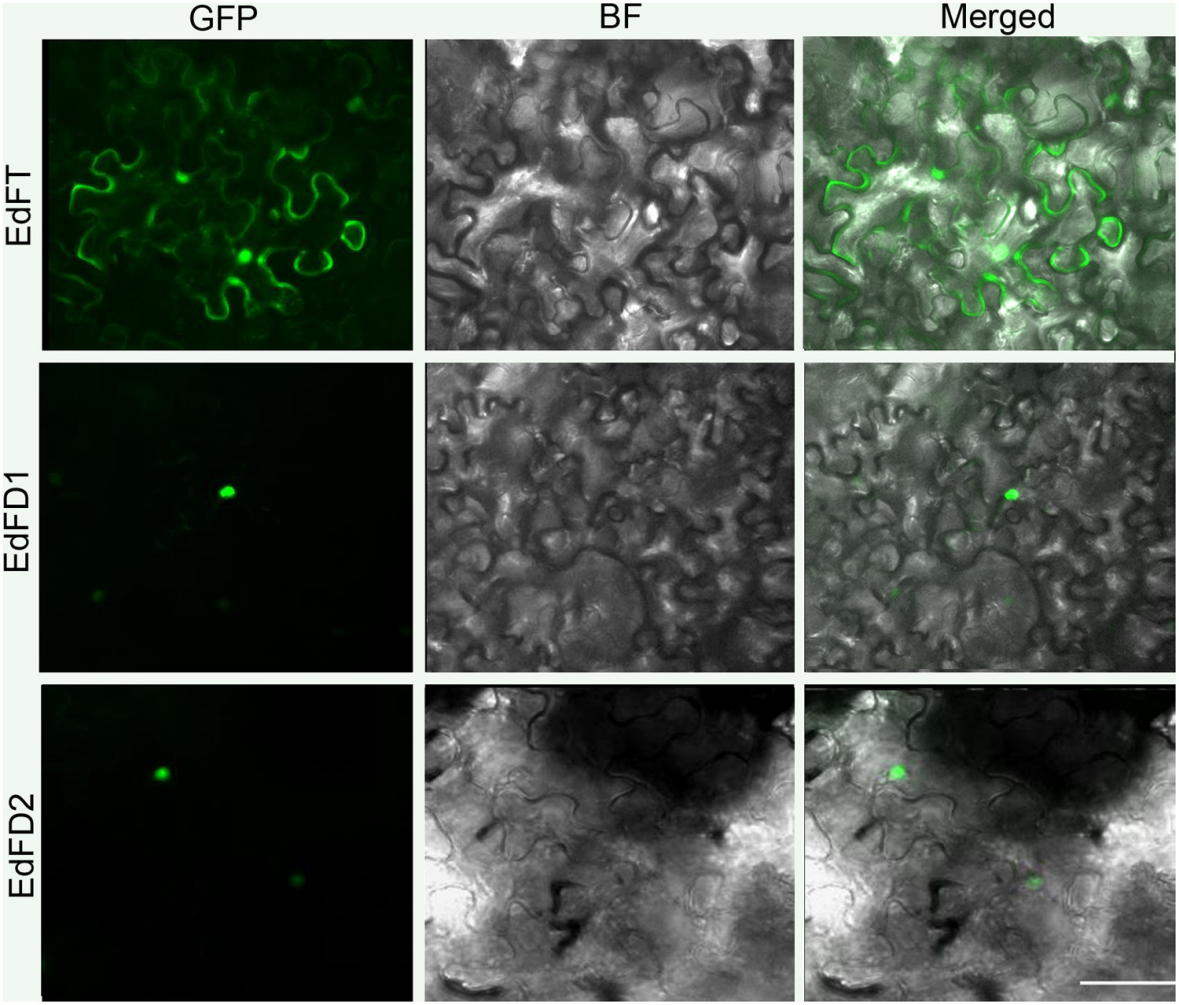
**Subcellular localization of EdFT and EdFDs.** GFP, GFP fluorescence; BF, bright-field; Merged, merged image of GFP and BF. Bar represents 50 μm.

We further examined the subcellular localization of EdFT and EdFDs by fusing the green fluorescent protein (GFP) at their C-terminal regions. The resulting constructs driven by the 35S promoter were then transiently expressed in leaf epidermal cells of *N. benthamiana*. EdFT-GFP was localized in both the cytoplasm and nucleus, while EdFD1-GFP and EdFD2-GFP were only detected in the nucleus (**Figure 3**). These subcellular localization patterns are similar to those of FT and FD in *Arabidopsis* (Abe et al., 2005).

### *EdFT* and *EdFDs* Accelerate Flowering in *Arabidopsis*

**FIGURE 4:**
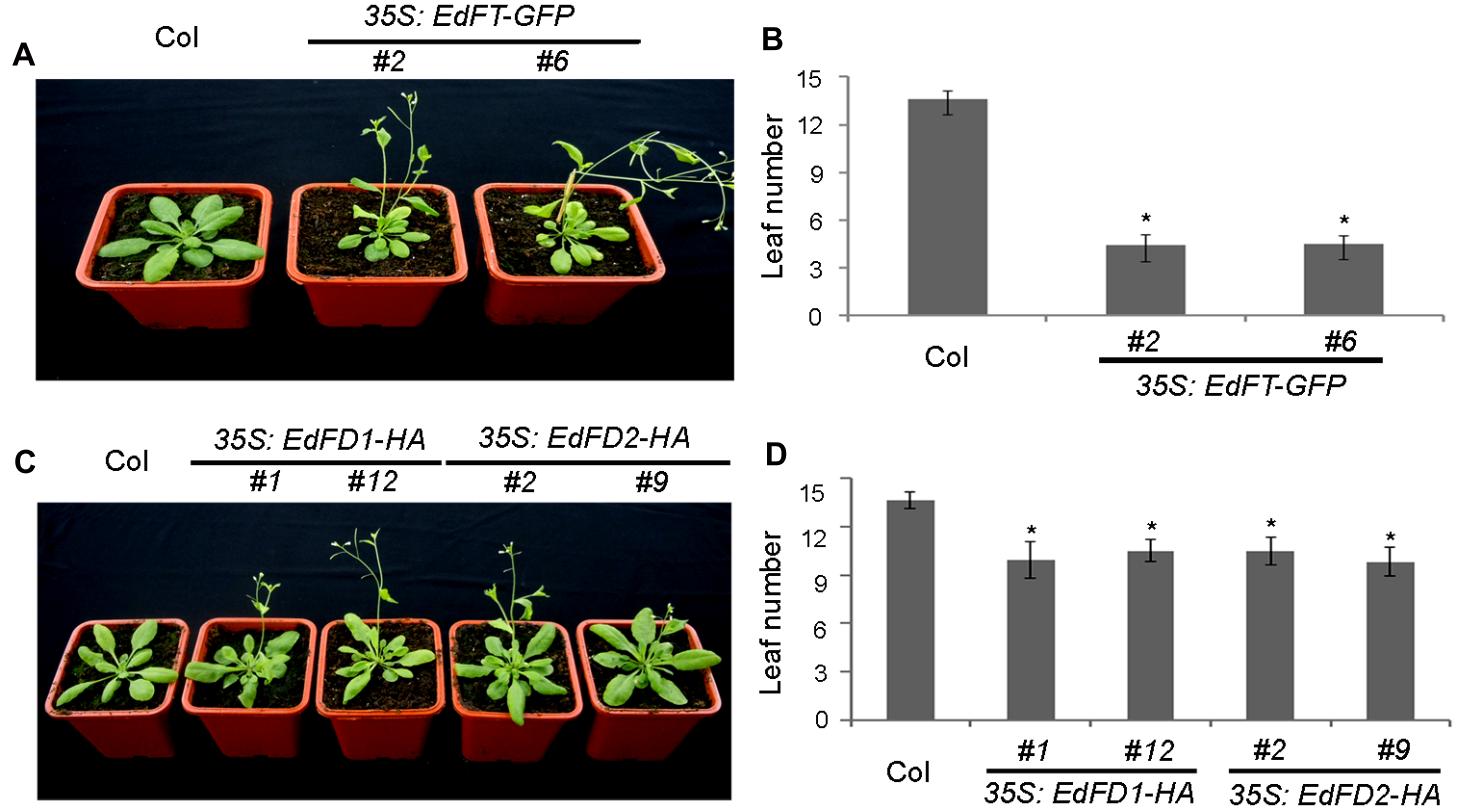
**Overexpression of *EdFT* and *EdFDs* in *Arabidopsis* accelerates flowering.** **(A)** *35S:EdFT-GFP* exhibits earlier flowering than wild type Col plants. **(B)** Rosette leaf number of Col and *35S:EdFT-GFP* transgenic lines. **(C)** *35S:EdFD1-HA* and *35S:EdFD2-HA* exhibit earlier flowering than wild type Col plants. **(D)** Rosette leaf number of Col and *35S:EdFD1/2-HA* transgenic lines. Asterisks indicate significant differences between Col and transgenic lines (*n* ≥ 10, *p* < 0.05, by Student’s *t*-test).

To further determine whether *EdFT* and *EdFDs* play a role in regulating flowering time, we generated *35S:EdFT-GFP*, 3*5S:EdFD1-HA*, and *35S:EdFD2-HA* transgenic *Arabidopsis* plants. We obtained more than 10 independent transgenic lines for each construct, and two lines of each genotype at the homozygous T3 generation with ectopic gene expression (**Supplementary Figure S2**) were selected for further investigation. *35S:EdFT-GFP*, *35S:EdFD1-HA*, and *35S:EdFD2-HA* transgenic lines displayed earlier flowering than Col wide-type plants under long-day conditions (**Figures 4A–D**). Wild-type plants flowered with 13–14 rosette leaves. In contrast, *35S:EdFT-GFP* transgenic lines flowered with only 3–5 rosette leaves, while *35S:EdFD1-HA* and *35S:EdFD2-HA* transgenic lines produced about 10 rosette leaves (**Figures 4B,D**). These results suggest that *EdFT*, *EdFD1*, and *EdFD2* play a conserved role in accelerating flowering in *Arabidopsis*.

### EdFT Interacts with EdFDs *In vivo*

**FIGURE 5:**
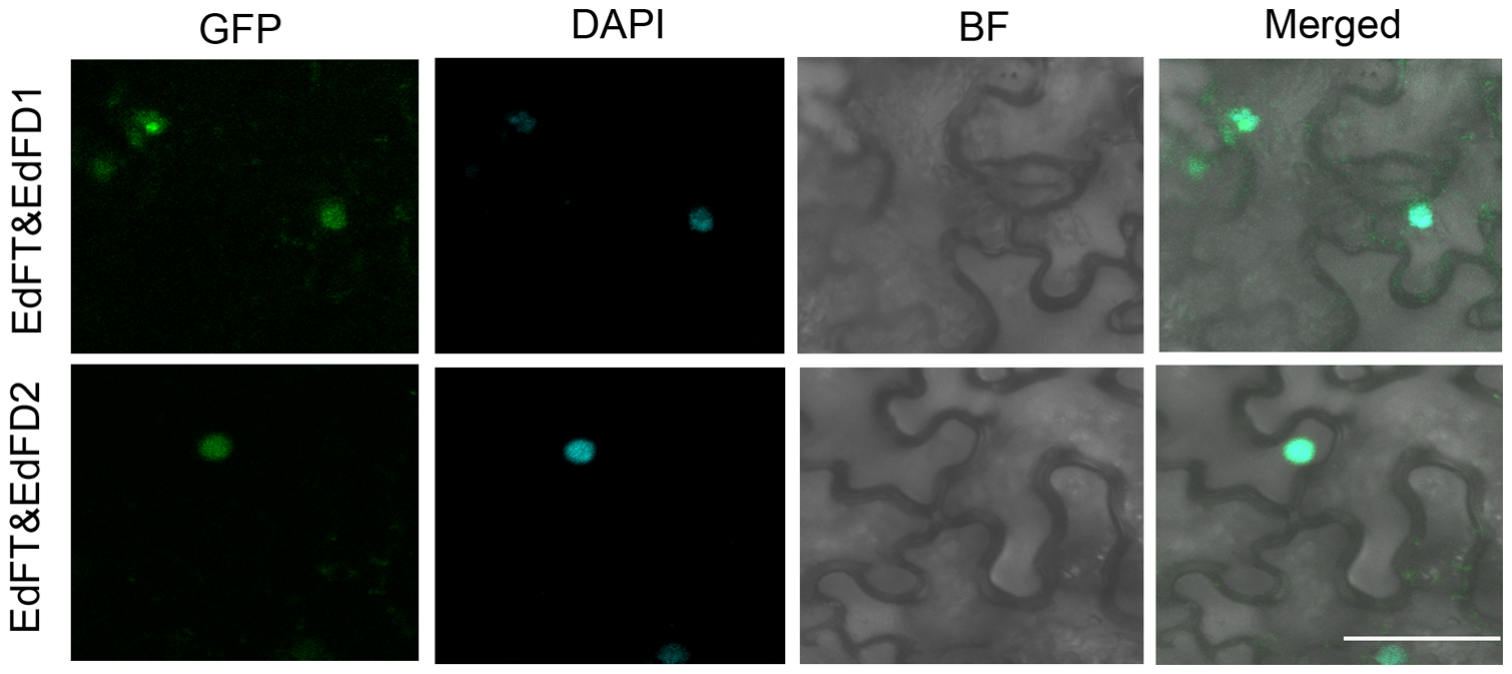
**Bimolecular fluorescence complementation (BiFC) analysis shows the protein interaction between EdFT and EdFDs.** EdFT&EdFD1, coexpression of *35S:EdFT-cYFP* and *35S:nYFP-EdFD1*; EdFT&EdFD2, coexpression of *35S:EdFT-cYFP* and *35S:nYFP-EdFD2*. GFP, GFP fluorescence; DAPI, fluorescence of 4, 6-diamino-2-phenylindol; BF, bright-field; Merged, merged image of GFP, DAPI, and BF. Bar represents 50 μm.

We further tested whether EdFT is able to interact with EdFD1 and EdFD2 as their counterparts in *Arabidopsis* using BiFC analysis. We constructed *35S:EdFT-cYFP* and *35S:nYFP-EdFD1* and *35S:nYFP-EdFD2* vectors. BiFC analysis clearly showed that the fluorescent signal could be detected in nuclei when EdFT-cYFP and nYFP-EdFD1 or nYFP-EdFD2 were co-expressed in *N. benthamiana* leaves (**Figure 5**), while the interaction was not observed in both EdFT-cYFP&nYFP and cYFP&nYFP-EdFD1/2 controls (**Supplementary Figure S3**). These results demonstrate the direct interaction of EdFT and EdFD1/2 in the nuclei of living plant cells.

## Discussion

In this study, we have isolated loquat genes, *EdFT*, *EdFD1* and *EdFD2*, from *E. deflexa* Nakai f. *koshunensis*. Sequence analysis of the deduced amino acids indicates their potential functional conservation with other reported orthologs in different flowering plants (**Figure 1** and **Supplementary Figure S1**). *EdFT* is expressed in flower buds, flowers and fruits, while *EdFD1* and *EdFD2* are expressed highly in leaf buds and flower buds (**Figure 2**). EdFT interacts with two EdFDs in the nucleus (**Figure 5**) in *N. benthamiana*. Furthermore, ectopic expression of these genes promotes *Arabidopsis* flowering (**Figure 4**). Taken together, these data suggest that EdFT and EdFDs are the loquat orthologs of *Arabidopsis* FT and FD proteins, respectively, and imply that there is a possibly conserved flowering regulatory mechanism involving these regulators in loquat.

In angiosperms, flowering is a vital transition from vegetative to reproductive growth. Proper timing of this process is a crucial factor that determines plant reproductive success. FT, as a component of long-sought florigen (Pennisi, 2007; Turck et al., 2008), has attracted a wide attention for investigating the floral transition. A large number of *FT*-like genes have been identified in various annual and perennial plants. Although most of the identified *FT* homologs have been shown to promote flowering, several *FT*-like genes have also been reported to likely evolve with new functions. In biennial cultivated sugar beet, flowering time is controlled by two *FT* genes (*BvFT1* and *BvFT2*) that exhibit antagonistic functions. *BvFT2* is required for flowering, whereas *BvFT1* represses flowering (Pin et al., 2010). In perennial poplar, expression of *FT1* and *FT2* is temporally and spatially separated. *FT1* initiates the transition from vegetative to reproductive growth, whereas *FT2* promotes the vegetative growth (Bohlenius et al., 2006; Hsu et al., 2011). Furthermore, *FT*-like genes have evolved to regulate stomata in *Arabidopsis* (Kinoshita et al., 2011), control tuberization in potato (Navarro et al., 2011), drive heterosis for yield in tomato (Krieger et al., 2010), mediate seasonal growth cessation and bud set in poplar (Bohlenius et al., 2006), and affect leaf and fruit development in apple (Mimida et al., 2011). In loquat, based on the transcriptome analysis of *E. deflexa* Nakai f. *koshunensis* leaves (unpublished data), only one *FT* homolog has so far been identified. In this study, ectopic expression of this *FT* ortholog implies a similar function in regulating flowering in loquat. In addition, high expression of *EdFT* in fruits implies that *EdFT* might also be engaged in regulating fruit development in loquat, which might be investigated in future studies. In the work, we cloned and characterized two orthologous *EdFD*s. These two EdFDs both could interact with EdFT in the nucleus, and promote flowering when they were ectopically expressed in *Arabidopsis*. However, whether these two EdFDs accelerate flowering through distinct modes, or have distinct roles in the interaction with EdFT, is needed to be elucidated in further study.

Flowering is influenced by multiple environmental cues, such as day length (photoperiod) and temperature (Andres and Coupland, 2012; Song et al., 2013), which mostly regulate flowering through FT. In *Arabidopsis*, long-day conditions induce B-box transcriptional factor CONSTANS (CO) to directly bind to the *FT* promoter to initiate flowering (Wenkel et al., 2006; Tiwari et al., 2010). The CO/FT regulatory model is probably conserved in most flowering plants (e.g., rice and poplar; Hayama et al., 2003; Bohlenius et al., 2006). Temperature, including the winter temperature and ambient temperature (Andres and Coupland, 2012; Song et al., 2013), is another key environmental signal that affects flowering time via other transcription factors in either the vernalization or the thermosensory pathway. In the vernalization pathway, FLOWERING LOCUS C (FLC), a MADS-box transcription factor that directly suppresses *FT* expression (Helliwell et al., 2006), is epigenetically silenced after a prolonged period of cold in winter, thus derepressing *FT* to initiate flowering (Swiezewski et al., 2009; Heo and Sung, 2011; Song et al., 2012). In the thermosensory flowering pathway, recent studies have shown a bHLH transcription factor PHYTOCHROME-INTERACTING FACTOR 4 (PIF4), a MYB transcription factor EARLY FLOWERING MYB PROTEIN (EFM), and two MADS-box transcription factors FLOWERING LOCUS M (FLM) and SHORT VEGETATIVE PHASE (SVP) play important roles in regulating flowering time through regulating *FT* expression in response to ambient temperature (Kumar et al., 2012; Lee et al., 2013; Pose et al., 2013; Yan et al., 2014). Based on observation of loquat growth in our germplasm resource preservation garden and some other provinces (e.g., Yunnan) in China, we have found that loquat can adjust appropriate flowering time after being transplanted. For example, *E. deflexa* Nakai f. *koshunensis* used in this study flowers in autumn–winter (October–January) in our garden, where as it flowers in spring (March) in the original place (Taiwan). The plasticity of various flowering patterns exhibited by loquat implies their ability of adjusting their responses to new environment signals to acclimatize themselves for the successful reproduction in new cultivation plots. In agreement with extensive studies in various flowering plants showing that flowering signal pathways mostly converge on the floral integrators *FT* and its orthologs, our study suggests that the *FT* ortholog in loquat could similarly play a critical role in mediating loquat response to various environmental signals. Further investigation of loquat orthologs of *FT* upstream regulators, such as CO, FLC, PIF4, EFM, FLM, or SVP transcription factors, could shed new light on flowering mechanisms in loquat.

## Conclusion

We characterized one *FT*- and two *FD*-like genes in loquat (*E. deflexa* Nakai f. *koshunensis*). And this study showed they were the functional homologs of *Arabidopsis* FT and FD, respectively. The *EdFT* and *EdFD*s promoted flowering in the ectopically expressed *Arabidopsis*. However, whether other FT orthologs exist in loquat? Do the two *EdFD*s accelerate flowering through distinct modes, or have distinct roles in the interaction with EdFT? And how do the environmental factors influence loquat flowering through *EdFT*? The further investigation will help unravel more details underlying these issues, and will facilitate in understanding the unique mechanism of loquat flowering time regulation.

## Author Contributions

LZ, SL, and YG designed and performed research. LZ, HY, SL, and YG wrote the paper.

## Conflict of Interest Statement

The authors declare that the research was conducted in the absence of any commercial or financial relationships that could be construed as a potential conflict of interest.
