# Supplementary material for: Molecular Characterization of FT and FD Homologs from Eriobotrya deflexa Nakai forma koshunensis
Source: Front Plant Sci. 2016 Jan 22;7:8. doi: 10.3389/fpls.2016.00008 (PMC4722113; doi:10.3389/fpls.2016.00008)
Supplement: Supplementary file 1 [file Data_Sheet_1.DOCX]

***Supplementary Material***

**Molecular Characterization of *FT* and *FD* homologs** **from** ***Eriobotrya deflexa* Nakai forma *koshunensis***

***Ling Zhang^1^, Hao Yu^2^, Shunquan Lin^1*^ and Yongshun Gao^1^****

**** Correspondence:***

*Corresponding Author: loquat@scau.edu.cn; yongshungao@163.com*

**Table S1. Primers used for gene cloning.**

| Primer name | Sequence (5’-3’) |
| --- | --- |
| *EdFT*-FP | ATGCCTAGGGATAGGGACCC |
| *EdFT*-RP | TTATCTTCTCCTTCCACCGG |
| *EdFD*-FP | ATGTTGTCATCAACAGGTAG |
| *EdFD*-RP | TCAAAATGGAGCTGTTGATG |

**Table S2. Primers used for vector construction.**

| Vector Name | Primer name | Sequence (5’-3’) |
| --- | --- | --- |
| *35S:EdFT-GFP* | *EdFT*-EcoRI | CGGAATTCATGCCTAGGGATAGGGAC |
|  | *EdFT*-SpeI | GGACTAGTTCTTCTCCTTCCACCGGA |
| *35S:EdFD1-HA/* | *EdFD1*-EcoRI | CGGAATTCATGTTGTCATCAACAGG |
| *35S:EdFD1-GFP* | *EdFD1*-SpeI | GGACTAGTAAATGGAGCTGTTGATG |
| *35S:EdFD2-HA/* | *EdFD2*-EcoRI | CGGAATTCATGTTGTCATCAACAGGT |
| *35S:EdFD2-GFP* | *EdFD2*-SpeI | GGACTAGTAAATGGAGCTGTTGATGT |
| *35S:EdFT-cYFP* | *EdFT*-XhoI-BiFC | CCGCTCGAGGAATGCCTAGGGATAGGGAC |
|  | *EdFT*-KpnI-BiFC | CCGGTACCGTCTTCTCCTTCCACCGGA |
| *35S:nYFP-EdFD1* | *EdFD1*-XhoI-BiFC | CCGCTCGAGGAATGTTGTCATCAACAGG |
|  | *EdFD1*-KpnI-BiFC | CCGGTACCGAAATGGAGCTGTTGATG |
| *35S:nYFP-EdFD2* | *EdFD2*-XhoI-BiFC | CCGCTCGAGGAATGTTGTCATCAACAGGT |
|  | *EdFD2*-KpnI-BiFC | CCGGTACCGAAATGGAGCTGTTGATGT |

**Table S3. Primers used for the analysis of gene expression by qPCR and RT-PCR.**

| Primer name | Sequence (5’-3’) |
| --- | --- |
| *EdACT4*-P1 | GGCATTCACGAAACCACT |
| *EdACT4*-P2 | CCACCTTGATCTTCATACTG |
| *EdFT*-P1 | GTTGTTGGACGAGTGGTAG |
| *EdFT*-P2 | TAACCTCTTTATTGCCGTAG |
| *EdFD1*-P1 | CCTCTTCCTCCATCCTCC |
| *EdFD1*-P2 | AGCAGCCAAGTTCTCAAAT |
| *EdFD2*-P1 | ATCCTGGCTCAGAGTTTCA |
| *EdFD2*-P2 | CTCCATCGGAATTGCTGT |
| *AtTUB2*-P1 | ATCCGTGAAGAGTACCCAGAT |
| *AtTUB2*-P2 | AAGAACCATGCACTCATCAGC |


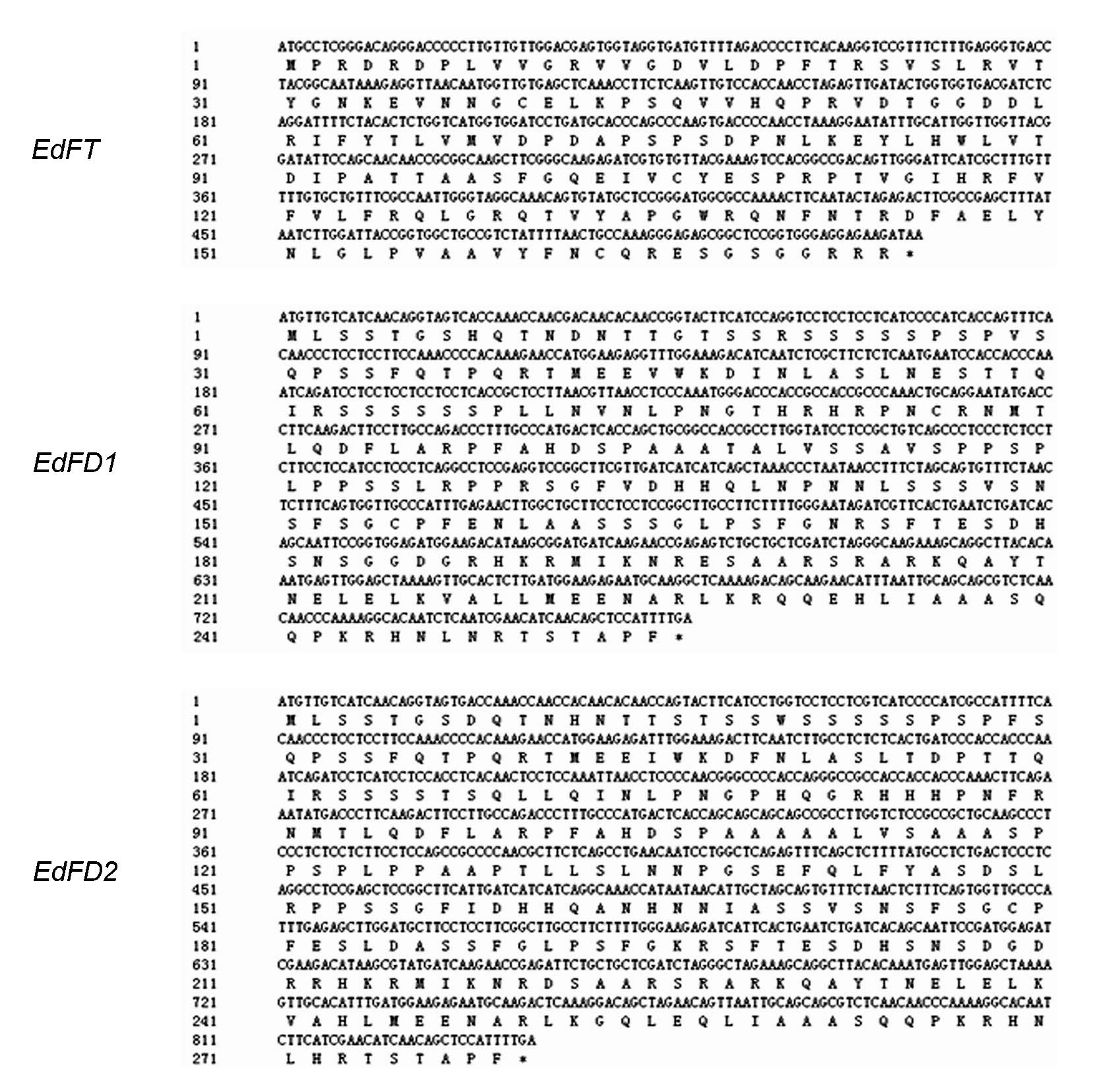


**Figure S1. The nucleotide and deduced protein sequences of *EdFT*, *EdFD1* and *EdFD2* genes.**


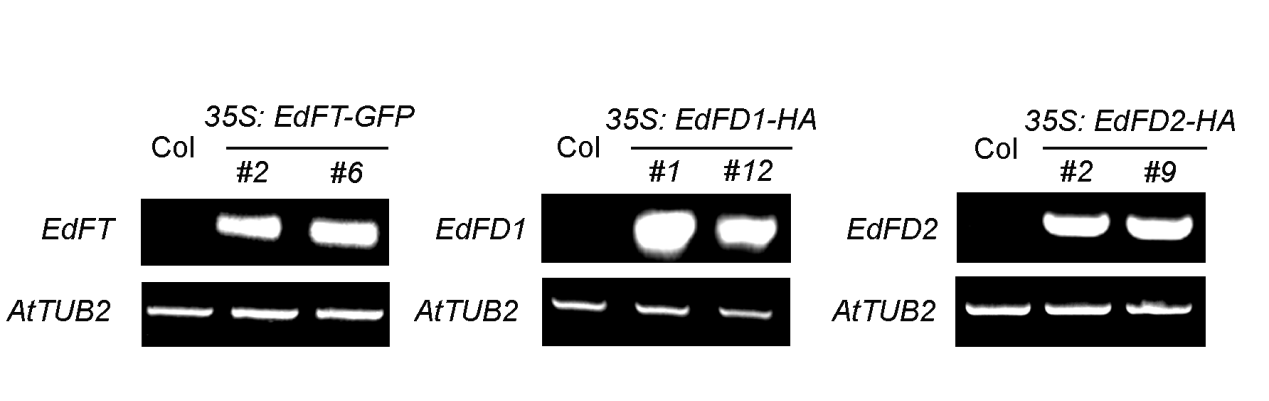


**Figure S2. *EdFT,* *EdFD1* and *EdFD2* highly expressed in seven-day-old transgenic *Arabidopsis* seedlings, respectively.** *Arabidopsis TUB2* (*AtTUB2*) severed as an internal control.


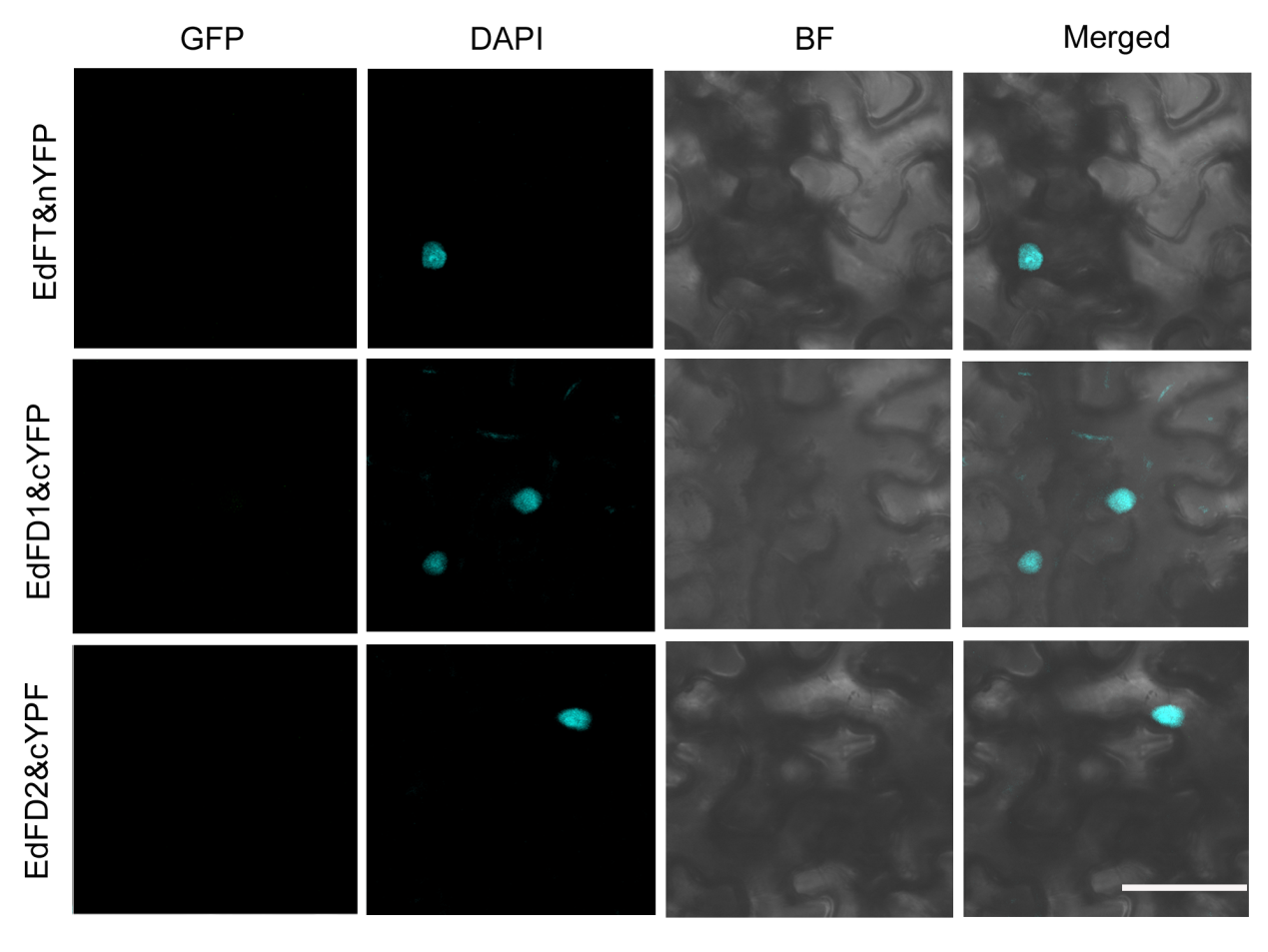


**Figure S3. Negative control of BiFC assay.** EdFT& nYFP, coexpression of *35S:EdFT-cYFP* and *35S:nYFP*; EdFD1&cYFP, coexpression of *35S:nYFP-EdFD1* and *35S:cYFP*; EdFD2&cYFP, coexpression of *35S:nYFP-EdFD2* and *35S:cYFP*. GFP, GFP fluorescence channel; DAPI, fluorescence of 4, 6-diamino-2-phenylindol; BF, bright-field; Merged, merged image of GFP, DAPI and BF. Bar represents 50 μm.
